# CD4+ Cells Regulate Fibrosis and Lymphangiogenesis in Response to Lymphatic Fluid Stasis

**DOI:** 10.1371/journal.pone.0049940

**Published:** 2012-11-20

**Authors:** Jamie C. Zampell, Alan Yan, Sonia Elhadad, Tomer Avraham, Evan Weitman, Babak J. Mehrara

**Affiliations:** The Division of Plastic and Reconstructive Surgery, Department of Surgery, Memorial Sloan-Kettering Cancer Center, New York, New York, United States of America; Universidade de Sao Paulo, Brazil

## Abstract

**Introduction:**

Lymphedema is a chronic disorder that occurs commonly after lymph node removal for cancer treatment and is characterized by swelling, fibrosis, inflammation, and adipose deposition. Although previous histological studies have investigated inflammatory changes that occur in lymphedema, the precise cellular make up of the inflammatory infiltrate remains unknown. It is also unclear if this inflammatory response plays a causal role in the pathology of lymphedema. The purpose of this study was therefore to characterize the inflammatory response to lymphatic stasis and determine if these responses are necessary for the pathological changes that occur in lymphedema.

**Methods:**

We used mouse-tail lymphedema and axillary lymph node dissection (ANLD) models in order to study tissue inflammatory changes. Single cell suspensions were created and analyzed using multi-color flow cytometry to identify individual cell types. We utilized antibody depletion techniques to analyze the causal role of CD4+, CD8+, and CD25+ cells in the regulation of inflammation, fibrosis, adipose deposition, and lymphangiogenesis.

**Results:**

Lymphedema in the mouse-tail resulted in a mixed inflammatory cell response with significant increases in T-helper, T-regulatory, neutrophils, macrophages, and dendritic cell populations. Interestingly, we found that ALND resulted in significant increases in T-helper cells suggesting that these adaptive immune responses precede changes in macrophage and dendritic cell infiltration. In support of this we found that depletion of CD4+, but not CD8 or CD25+ cells, significantly decreased tail lymphedema, inflammation, fibrosis, and adipose deposition. In addition, depletion of CD4+ cells significantly increased lymphangiogenesis both in our tail model and also in an inflammatory lymphangiogenesis model.

**Conclusions:**

Lymphedema and lymphatic stasis result in CD4+ cell inflammation and infiltration of mature T-helper cells. Loss of CD4+ but not CD8+ or CD25+ cell inflammation markedly decreases the pathological changes associated with lymphedema. In addition, CD4+ cells regulate lymphangiogenesis during wound repair and inflammatory lymphangiogenesis.

## Introduction

Lymphedema is a chronic disorder that is characterized by progressive tissue swelling and fat deposition secondary to congenital defects, infections, or injury to the lymphatic system. In its most advanced forms, lymphedema results in massive changes in the extremities referred commonly to as elephantiasis. Although the most common cause of lymphedema worldwide is parasitic infections with nematodes such as *Wuchereria bancrofti*, these infections are rarely seen in developed countries where lymphedema occurs most commonly after cancer surgery. [1] In these cases, patients develop lymphedema after direct injury to the lymphatic system resulting from lymph node dissection or secondarily from wide skin excision and radiation therapy. It is estimated that as many as 1 in 3 women treated with axillary lymph node dissection for breast cancer develop lymphedema.[2] Lymphedema is also common in other solid malignancies occurring in nearly 1 in 8 patients treated for a variety of tumors. [3].

The lack of a clear understanding of the pathology of lymphedema has served as a significant barrier to the development of effective, targeted treatments or preventative options for this disabling complication. Instead, current treatments are palliative in nature with a goal of preventing disease progression and decreasing symptoms rather than curing the underlying pathology. The fact that lymphedema in most cases develops 8–12 months after surgery (rather than immediately following lymph node dissection) [4] suggests that lymphatic injury is merely an initiating event that is necessary for activation of cellular and molecular changes that over time lead to chronic tissue edema, inflammation, fibrosis, and fat deposition. It remains unknown, however, if the key histological features of lymphedema such as inflammation and fibrosis play a causal role in this pathology or if these changes simply reflect worsening pathology. Similarly, although previous reports have demonstrated that patients with secondary lymphedema have high concentrations of lymphocytes in peripheral lymph, increased density of Langerhans cells and class II antigen expression in the skin, and increased granulocyte margination in lymphedematous tissues, [5], [6], [7], [8] the precise cellular response to lymphatic fluid stasis and chronic lymphedema remain unknown. This is important since recent studies have shown critical roles for inflammatory cells in the regulation of fibrosis, lymphangiogenesis, and adipose tissue deposition in other disorders. [9], [10], [11], [12] Therefore, understanding how lymphatic fluid stasis regulates these responses acutely, and more importantly, how these responses are coordinated chronically is an important first step in developing targeted treatments that can block initiation or progression of the pathological consequences of lymphatic injury.

With these goals in mind, the purpose of this study was to determine how lymphatic stasis regulates tissue inflammatory changes. In addition, we sought to determine if these chronic inflammatory responses play a pathological role in lymphatic dysfunction, fibrosis, and adipose deposition in lymphedema. We show that specific T cell subpopulations are a critical component of the subacute and chronic inflammatory cell response to lymphatic fluid stasis. In addition, using antibody depletion studies, we show that T-helper cell inflammatory responses are necessary for fibrosis, lymphatic dysfunction, subcutaneous fat deposition, and chronic inflammation occurring in response to sustained lymphatic fluid stasis. Our findings are important because we show for the first time that lymphedema results in characteristic inflammatory responses. Further, we show that these responses play a role in the pathology of lymphedema rather than simply reflecting worsening pathology.

## Methods

### Animal Models

Adult female (10–12 week old) wild-type C57B6 or transgenic C57B6 mice deficient in CD4+ cells (CD4KO; Jackson Labs, Bar Harbor, ME) were used and all studies were performed according to IACUC standards and approved animal protocols at Memorial Sloan-Kettering Cancer Center. A total of 8–10 animals were used for each group/experiment.

In order to study the effects of sustained, severe lymphatic stasis on inflammatory responses, we used a well-described mouse tail model of lymphedema in which the superficial and deep lymphatic system of the tail are disrupted by excising a 3 mm portion of the skin and microsurgically ligating the deep lymphatic channels that run along the lateral tail veins. [13], [14], [15], [16] Our group, and others, have previously shown that this model results in sustained lymphedema of the distal tail, severe impairment in lymphatic function, and histological features of clinical lymphedema (e.g. chronic inflammation, adipose deposition, fibrosis) for at least 10 weeks postoperatively. [13], [14], [15], [16] Control animals underwent circumferential tail skin incision but did not undergo superficial or deep lymphatic system disruption. Lymphatic function, flow cytometry, and histological analysis were performed 6 weeks after surgery as described below.

We also used a mouse model of axillary lymph node dissection to study the consequences of lymphadenectomy and disruption of the deep lymphatic system. We have previously shown that similar to the clinical scenario, axillary lymph node dissection (ALND) in mice results in minor though significant increases in limb volume enabling us to study acute tissue changes that occur in response to lymphatic stasis. [17] Experimental mice underwent ALND dissection using a 1 cm axillary incision and all identifiable axillary lymph nodes were removed. Control animals underwent axillary skin incision without lymph node removal.

### Flow Cytometry

A 1 cm portion of tissue was harvested 1 cm distal to the site of axillary lymph node dissection or 1.5 cm distal to the site of tail skin excision. Flow cytometry and analysis was performed using a modification of our previously reported methods. [18] Briefly, skin and subcutaneous tissues were stripped from the underlying tissues and digested at 37°C for 30 min in phosphate-buffered saline (PBS) solution of collagenase D (0.2 mg/ml), DNAse I (0.1 mg/ml), Dispase (0.1 mg/ml), and 2% fetal calf serum (all from Sigma Aldrich, St. Louis, MO). Tissue digests were then filtered and resuspended as a single cell suspension in PBS/2%FCS/Sodium azide solution for flow cytometry analysis. Splenocytes were isolated simultaneously by rupturing spleens, lysing red blood cells, and filtering to achieve single cell suspensions. Cells were blocked at 4° with Fc block (CD16/CD32, eBiosciences (San Diego, CA) to block endogenous Fc receptors. Single stains were performed on splenocytes for optimization of cytometer settings. Cell suspensions isolated from peripheral tissues were then stained using fluorophore-conjugated antibodies for the following cell surface markers: CD45, CD4, CD8, TCRB, CD25, F4/80, CD11b, CD11c, Ly6c, Ly6g, B220 (all antibodies from Biolegend, San Diego, CA), and NK T cell-specific Tetramer (PBS57–CD1d tetramers were obtained from the US National Institutes of Health Tetramer Core Facility). Alternatively, cell suspensions were Fc-blocked followed by fixation, permeabilization overnight at 4°C, and stained for the intracellular marker FoxP3 (eBiosciences ) and cell surface markers CD4 and CD25 (Biolegend).

Cell suspensions were analyzed using an LSR II flow cytometer (BD Biosciences, San Jose CA) with BD FACSDiva software and subsequent data analysis performed using FlowJo software (Tree Star, Ashland, OR). Cell populations were analyzed and defined using the cell surface markers listed in Table 1 using 5–7 animals/group/experiment with each experiment performed in triplicate.

**Table 1:** Cell surface markers for identification of leukocyte cell types.

Cell Type	Surface Marker
Leukocytes	CD45^+^
Mature T-helper cells	CD45^+^/CD4^+^/TCRB^+^
Mature T-cytotoxic cells	CD45^+^/CD8^+^/TCRB^+^
T-regulatory cells	CD4^+^/D25^+^/Foxp3^+^
Natural killer T cell	NKT; CD4^+^/TCRB^+^/Tetramer^+^
Macrophage	CD45^+^/CD11b^+^/F4/80^+^
Dendritic cell	CD45^+^/CD11c^+^/MHC II^hi^
Neutrophil	CD45^+^/CD11b^+^/Ly6g^hi^
Monocyte	CD45^+^/CD11b^+^/Ly6c^hi^
B cell	CD45^+^/B220^+^

### Depletion Experiments

Depletion experiments were performed using the tail model of lymphedema as described above. Following tail skin and lymphatic vessel excision, animals were allowed to recover for 2 weeks and then randomized to experimental or control groups (n = 8–10/group). Experimental animals were depleted of CD4, CD8, or CD25 cells using intraperitoneal administration of monoclonal neutralizing antibodies (all from Bio-X-Cell, Lebanon, New Hampshire) at a concentration of 10 ug/g every 5 days for a total of 4 weeks (i.e. 6 weeks postoperatively) as previously described. Control animals were treated with non-specific isotype control antibodies delivered at the same dose and timing. Adequacy of depletion was confirmed by flow cytometry analysis of splenic single cell suspensions comparing experimental and control animals at the time of sacrifice (6 weeks postoperatively).

### Tail Volume, Lymphoscintigraphy, Microlymphangiography

Tail lymphedema was monitored weekly using multiple digital caliper tail circumference measurements distal to the zone of lymphatic injury and calculation of tail volumes using the truncated cone formula as previously described. [15] Lymphoscintigraphy was performed as previously described to quantify lymphatic flow by injecting 50 ml of filtered technetium (Tc^99 m^) sulfur colloid in the distal tail and analyzing decay adjusted uptake in the sacral lymph nodes using an X-SPECT camera (Gamma Medica, Northridge, CA) and region-of-interest analysis using ASIPro software (CTI Molecular Imaging, Knoxville, TN). [15] To analyze lymphatic architecture and calculate interstitial fluid flow, we performed microlymphangiography using our previously published techniques. [14] Briefly, fluorescein isothiocyanate (FITC)-conjugated dextran (2,000 kDa, 10 mg/ml, Invitrogen, Carlsbad, CA) was injected under constant anatomic pressure in the distal tail and then visualized 15 minutes later at fixed 10 mm intervals along the tail using the Lumar Stereoscope (Carl Zeiss Inc, Peabody, MA) and Metamorph imaging software (Molecular Devices, Sunnyvale CA). Exposure, gain, magnification, and body temperature were kept constant and the animal was anesthetized to prevent motion artifact. Uptake of FITC-dextran was expressed as a ratio of mean pixel intensity of regions proximal relative to those distal to the surgical site 15 minutes post-injection.

### Lymph Node lymphangiogenesis

Lymph node lymphangiogenesis was used as previously described to determine how CD4 cells regulate inflammatory lymphangiogenesis independent of wound healing. Briefly, a mixture of complete Freund’s adjuvant (Sigma) and ovalbumin (1∶1) was emulsified and injected in the hind paws of adult female wild-type C57B6 mice and popliteal lymph nodes were harvested 7 days later. Experimental animals (n = 8) were depleted of CD4+ cells using monoclonal neutralizing antibodies beginning 3 days prior to CFA/OVA injection as outlined above while control animals (n = 8) were treated with an equivalent dose of non-specific isotype control antibodies. To confirm our findings with CD4 depletion, we performed identical experiments in wild-type C57B6 mice (n = 8) and compared them with transgenic C57B6 mice deficient in CD4 cells (CD4KO; n = 8; Jackson labs).

### Immunohistochemistry and Histological Analysis

Tail tissues were fixed in 4% paraformaldehyde and decalcified in Immunocal prior to paraffin embedding. Lymph nodes were embedded in OCT medium (Sigma-Aldrich, St. Louis, MO) and frozen at −80°C; 5 um sections were prepared from all tissues for analyses.

Immunohistochemical staining using horseradish-peroxidase based staining techniques were performed according to our established techniques [14]. Briefly, paraffin-embedded tissues were rehydrated, and antigen unmasking was performed using boiling sodium citrate (Sigma). Endogenous peroxidase activity was quenched and non-specific binding was blocked with 2% BSA/20% animal serum. Tissues were incubated with primary antibody overnight and antibody staining was visualized using horseradish-peroxidase conjugated secondary antibodies and development with diaminobenzamine complex (DAB; Vector, Burlingame, CA). Primary antibodies used for immunohistochemical stains included collagen I, collagen III, podoplanin, CD4, F4/80, CD45 (all from Abcam, Cambridge, MA), and vascular endothelial growth factor-C (VEGF-C; Novus biological, Littleton, CO). All secondary antibodies were obtained from Vector Laboratories. Sections were analyzed using brightfield microscopy and regions of interest were scanned using a Mirax slide scanner (Zeiss, Munich, Germany). Cell counts were performed on high-powered sections from a minimum of 4–6 animals per group and 4–5 hpf/animal by 2 blinded reviewers.

Immunofluorescent stains were performed as described above, excluding peroxidase quenching steps. Primary antibody to LYVE-1 (R&D Systems, Minneapolis, MN) and TRITC-conjugated secondary antibody were utilized for staining. For frozen lymph node sections, slides were fixed briefly in acetone followed by blocking and staining techniques as described above. Lymphatic vessel density was determined using Metamorph Scanner and Metamorph Offline software (Molecular Devices, Sunnyvale CA) as previously described. [19].

Subcutaneous tissue thickness analysis was performed in histological cross-sections located 1.5 cm distal to the surgical site by blinded reviewers (n = 6–8 per group). The distance from the basal layer of the epidermis to deep fascia was analyzed in 4 standardized regions per section. Lymphatic vessel area was measured as previously described. [14] Briefly, the widest radius of individual lymphatic vessels identified by podoplanin or LYVE-1 staining in histological cross sections was used to calculate lymphatic vessel area. Tissue fibrosis was assessed using our previously published methods for Sirius red quantification (scar index) as well as by immunohistochemical staining for type I and type III collagen. [14] Collagen deposition was quantified as a ratio of positively stained dermis and subcutaneous tissues within a fixed threshold to total tissue area using Metamorph Offline software.

### Protein Analysis

Tail tissues or lymph nodes were lysed (Thermofischer Scientific, Waltham, MA) and 20–30 mg of pooled protein (n = 3–5 animals) was used for western blot analysis using our previously published methods. [14] We analyzed the expression of interferon-gamma (IFN-y), interleukin-4 (IL-4; both from Abcam), FoxP3, Tbet, GATA3, (all from Santa Cruz Biotechnology, Santa Cruz, CA), type I collagen, type III collagen, alpha-smooth muscle actin (a-SMA), E-cadherin, phosphorylated SMAD-3 (pSMAD3; all from Abcam), TGFB-1 (Santa Cruz), VEGF-A (Abcam), and VEGF-C (Novus Biologic). Equal loading was ensured with actin (Abcam) and band density was determined using ImageJ analysis (http://rsbweb.nih.gov/ij/). Normalized expression was determined as a ratio relative to controls. For ELISA, 20–30 mg of protein from pooled samples (n = 3–5 animals/group) was analyzed to quantify VEGF-A and VEGF-C according to the manufacturer’s directions (eBioscience, San Diego, CA). All experiments were performed in triplicate.

### Statistical Analysis

Student’s T-test was used to compare differences between 2 groups; paired T-tests were performed for all matched ALND and sham control samples. Multi-group comparisons were performed using 2-way analysis of variance (ANOVA) with Tukey-Kramer post-hoc test. Data are presented as mean ± standard deviation unless otherwise noted with p<0.05 considered significant.

## Results

### Chronic Lymphedema Results in a Mixed Inflammatory Cell Response

**Figure 1:**
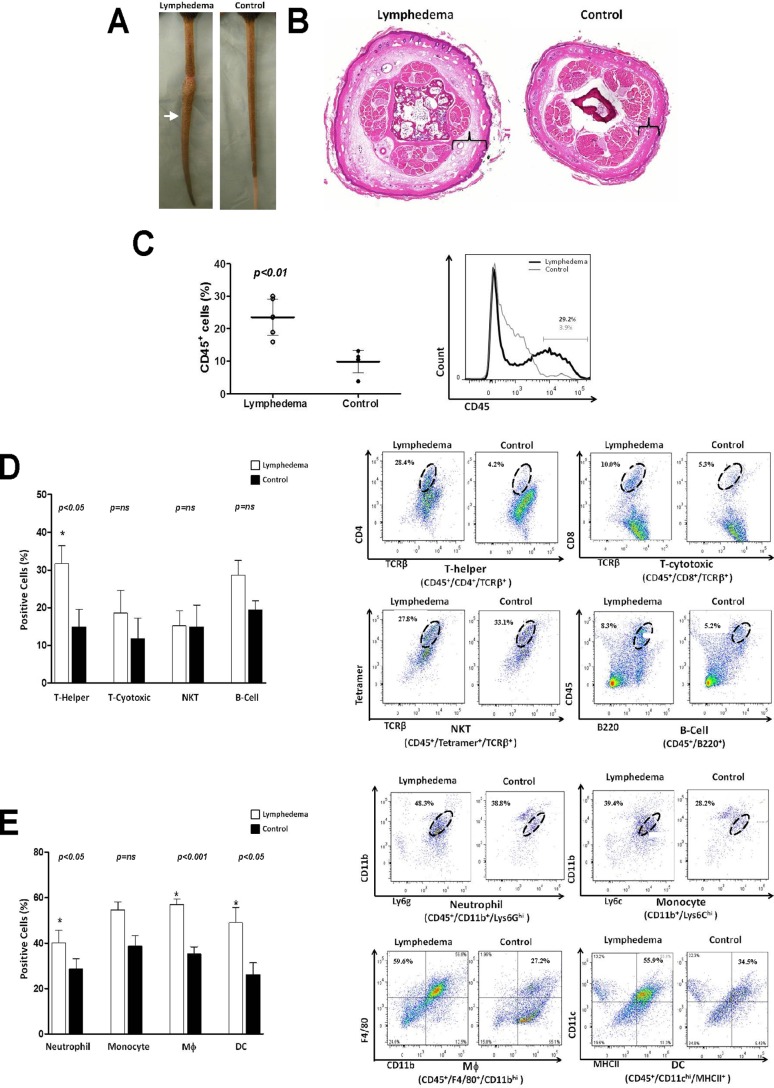
Chronic lymphedema results in a mixed inflammatory cell response. **A.** Photograph of mouse-tails 6 weeks after skin/lymphatic excision (lymphedema; left) or skin incision (control; right). **B.** Representative cross sectional histology of mouse tails comparing lymphedema (left) and control (right) mice 6 weeks after surgery. Cross sections were obtained 2 centimeters distal to the tail wound (arrow in figure A). Note subcutaneous fat deposition (brackets), dilated lymphatics, and inflammation in lymphedema section. **C.** Flow cytometry analysis for CD45+ cells in single cell suspensions prepared from tail tissue 2 cm distal to the wound of lymphedema or control mice (n = 5–7/group) 6 weeks after surgery. The percentage of CD45+ cells as a function of total cell population is shown. A representative histogram is shown to the right. **D., E.** Flow cytometry analysis of T-helper, T-cytotoxic, natural killer T cells (NKT), B cell (Figure D) and neutrophils, monocytes, macrophage, and dendritic cells (Figure E) in single cell suspensions of lymphedematous or control mice (n = 5–7/group) 6 weeks after surgery. Representative dot plots are shown to the right. Oval gates indicate double positive cell populations.

We used a well-described mouse-tail model of lymphedema to determine how chronic lymphatic stasis regulates tissue inflammation. As expected, mice that underwent tail skin and lymphatic excision demonstrated markedly increased tail volumes, tail contracture and fibrosis, and hyperkeratosis 6 weeks after surgery (**Figure 1A, B**). In contrast, control animals that underwent tail skin incision only had no swelling or abnormal changes at this time. Analysis of tissue digests harvested 1.5 centimeter distal to the zone of lymphatic injury also confirmed our previous observations [14] and demonstrated markedly increased numbers of CD45^+^ cells consistent with tissue inflammation when compared with controls (2.3 fold; **Figure 1C**). Multicolor flow cytometry analysis of these tissue samples demonstrated that the percentage of mature T-helper (CD45^+^/CD4^+^/TCRB^+^) was also dramatically increased in lymphedematous tissues (2.2 fold; **Figure 1D**). In contrast, we noted no significant differences in the percentage of mature cytotoxic T cells, NKT cells, or B Cells. Interestingly, and consistent with our previous reports, [17] we also noted significantly increased percentage of neutrophils (1.4 fold), macrophages (1.6 fold), and dendritic cells (1.9 fold) in lymphedematous tissues (**Figure 1E**).

### Axillary Lymph Node Dissection Results in T Cell Inflammation

In order to determine the type of inflammatory responses that are initiated by lymphatic injury prior to the onset of chronic lymphedema, we analyzed tissue inflammation in the upper extremity of mice that had undergone axillary lymph node dissection (ALND) or axillary skin incision without lymphatic injury 3 or 6 weeks after surgery. This approach was based on our previous studies demonstrating that: **1).** ALND in this model (similar to the clinical scenario) results in mild but significant edema of the upper extremity at 3 weeks postop and resolves by 6 weeks; **2.)** Tissue inflammatory responses to ALND are histologically evident beginning primarily 3 weeks after surgery. [17], [20].

**Figure 2:**
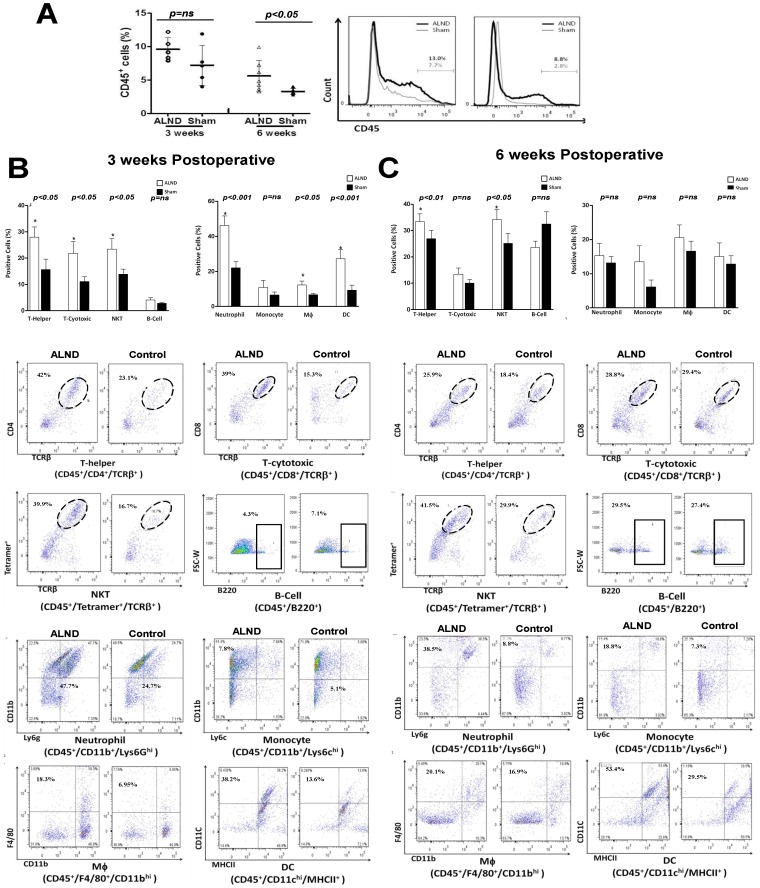
Axillary lymph node dissection results in a T cell inflammatory reaction. A. Flow cytometry analysis for CD45+ cells in single cell suspensions prepared from upper extremity soft tissues 1.5 cm distal to the axillary wound in animals treated with axillary lymph node dissection (ALND) or axillary incision without lymphadenectomy (sham; n = 5–7/group) 3 or 6 weeks after surgery. A representative histogram is shown to the right. **B**. Flow cytometry analysis of T-helper, T-cytotoxic, natural killer T cells (NKT), B cell (top panel) and neutrophils, monocytes, macrophage, and dendritic cells in single cell suspensions of upper extremity soft tissues harvested **3 weeks** after ALND or sham incision (n = 5–7/group). Representative dot plots are shown below. **C**. Flow cytometry analysis of T-helper, T-cytotoxic, natural killer T cells (NKT), B cell (top panel) and neutrophils, monocytes, macrophage, and dendritic cells in single cell suspensions of upper extremity soft tissues harvested **6 weeks** after ALND or sham incision (n = 5–7/group). Representative dot plots are shown below.

At the 3-week time point, we noted a mild (1.3 fold) but insignificant increase in the percentage of CD45+ leukocytes in animals treated with ALND as compared to controls (**Figure 2A**). This difference became more pronounced (1.8 fold) and significant by 6 weeks post operatively. Interestingly, when we analyzed inflammatory cell subtypes 3 weeks after surgery, we noted significant increases in the percentages of T cell subtypes (1.8 fold increase T-helper cells, 2 fold increase in cytotoxic T cells, and 1.7 fold increase in NKTs) as well as neutrophils (2.1 fold). However, B cells, monocytes, macrophages, and dendritic cells were little changed (**Figure 2B**). The increased percentage of T helper cells (1.3 fold) and NKTs (1.4 fold) persisted at the 6 week time point, however, differences between cytotoxic T cells and neutrophils became insignificant (**Figure 2C** **)**. Similarly, we found no significant differences in the percentage of neutrophils, monocytes, macrophages, or dendritic cells at this time point.

### CD4 Cell Depletion Reduces Lymphedema

Based on the finding that T cell inflammation is initiated by lymphatic injury (ALND) and persists in chronic lymphedema, we tested the hypothesis that these inflammatory reactions contribute to the pathology of lymphedema by performing antibody depletion studies. This hypothesis is supported by recent findings demonstrating that T cells can regulate lymphangiogenesis [11], [21], [22] and are known to play a role in fibrotic disorders and may therefore contribute to the pathologic fibrosis that is associated with chronic lymphedema.

**Figure 3:**
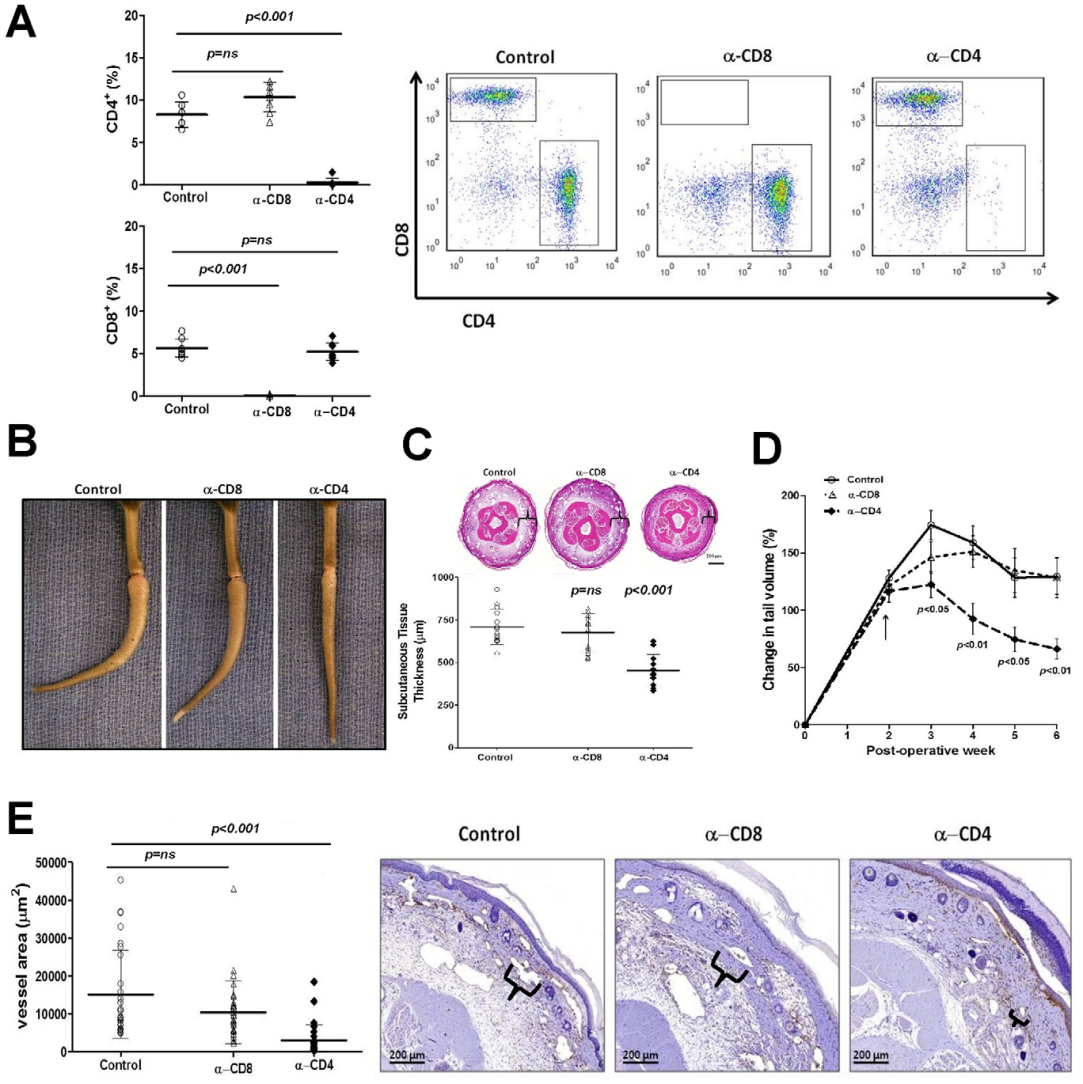
CD4 cell depletion reduces lymphedema. A. Flow cytometry analysis of splenic single cell suspensions from mice treated with isotype control antibodies or depleted of CD4+ cells (upper panel) or CD8+ cells (lower panel) using neutralizing antibodies (n = 5–7/group). Representative dot plots are shown to the right. **B.** Representative photograph of control, CD8+ cell depleted, or CD4+ cell depleted mice 6 weeks after tail superficial and deep lymphatic excision. Note near complete resolution of edema in CD4+ treated animals and loss of fixed tail contracture (“J” shape seen in control or CD8+ treated animals). **C.** Representative cross sectional histology and quantification of subcutaneous tissue thickness (brackets) in control, CD8+, and CD4+ depleted animals. **D.** Tail volumes in control, CD8+, or CD4+ depleted animals over the course of the experiment. CD4+ or CD8+ cell depletion was begun 2 weeks after surgery (arrow). **E.** Analysis of lymphatic vessel diameter (podoplanin+ vessels) in control, CD8+, or CD4+ depleted animals (left) and representative photomicrographs (right). Lymphatic vessel diameter is shown in brackets.

As expected, depletion of CD4 or CD8 cells with neutralizing antibodies was highly effective resulting in near complete absence of these cells in the spleen (**Figure 3A**). We chose to begin T cell depletion 2 weeks after tail skin and lymphatic excision based on our previous studies demonstrating that inflammatory reactions in this model first become manifest at this time point. [15] Interestingly, we found that depletion of CD4 cells resulted in marked decreases in tail lymphedema and reduced fibrotic tail curvature distal to the site of lymphatic disruption when compared to controls or animals treated with CD8 neutralizing antibodies (**Figure 3B**). These changes were reflected in significantly decreased tail volumes (1.9 fold) and subcutaneous tissue thickness of histological sections (1.5 fold) in CD4 depleted mice (**Figure 3C, D**). Histological sections demonstrated markedly decreased inflammation and subcutaneous edema and fat deposition in CD4 depleted animals (**Figure 3C**). In contrast, CD8 depletion had little effect. Changes in tail volume, subcutaneous adipose tissue deposition, and inflammation in CD4 depleted mice correlated with decreased podoplanin positive lymphatic vessel diameter (**Figure 3E**) suggesting that these animals have decreased lymphatic stasis because their capillary and collecting lymphatics are collapsed. [**16**] In contrast, control and CD8 depleted animals had large, dilated, ecstatic lymphatic vessels consistent with persistent lymphatic dysfunction. Taken together, these findings suggest that lymphatic stasis causes T cell inflammation and that CD4+ cells may contribute to the pathologic changes that ensue.

### CD4^+^ Cell Depletion Reduces Lymphedema Induced Chronic Inflammation

**Figure 4:**
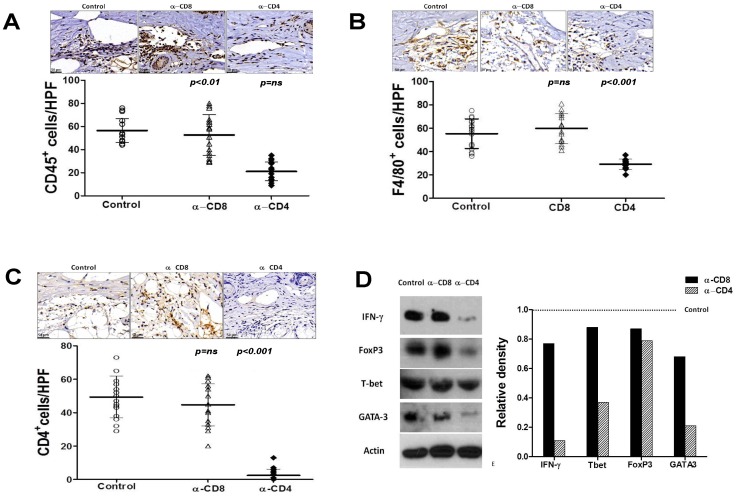
CD4^+^ cell depletion reduces lymphedema induced chronic inflammation. A, B, C. Representative photomicrographs of CD45 (figure A), F4/80 (figure B), and CD4 (figure C) immunohistochemical staining in tail tissues of control, CD8+, or CD4+ cell depleted animals 6 weeks after tail superficial and deep lymphatic excision. Quantification of cell numbers per high-powered field (hpf) are shown below for each cell type. **D.** Representative (of triplicate experiments) western blots from tail tissues for Th1 (IFN-y, Tbet), T-reg (FoxP3), and Th2 (Gata-3) markers in control, CD8+, and CD4+ depleted animals 6 weeks after tail superficial and deep lymphatic excision. Quantification of band density relative to controls (arbitrarily set at 1 and represented by dotted line) is shown to the right.

Since CD4 depletion after lymphatic injury resulted in decreased lymphedema, we hypothesized that CD4 cells may be responsible for regulating inflammatory changes in tissues exposed to lymphatic fluid stasis. To test this hypothesis, we analyzed inflammatory cell populations in the tissues located distal to the zone of lymphatic injury using immunohistochemistry in animals treated with CD4 or CD8 neutralizing antibodies. This analysis demonstrated that animals treated with CD4 neutralizing antibodies had significantly decreased numbers of infiltrating CD45^+^ leukocytes (2.5-fold), CD4^+^ cells (16.7-fold), and F4/80^+^ macrophages (2-fold; **Figures 4 A–C**).

Western blot analysis of whole tissue protein demonstrated marked decreases in expression of Th1 and Th2 markers in CD4 but not CD8 depleted animals as compared with controls (**Figure 4D**). For example, expression of Th1 markers IFN-y and T-bet were decreased by 9 and 2.7 folds, respectively. Similarly, expression of the Th2 marker Gata-3 was decreased by 4.8 fold. The expression of FoxP3, a marker of T regulatory cell differentiation was also slightly decreased (1.3 fold) in CD4 depleted animals. Together, these findings suggest that CD4 but not CD8 depletion after lymphatic injury decreases inflammatory responses to lymphatic stasis resulting in decreased overall inflammation, decreased numbers of T cells and macrophages, and decreased expression of both Th1 and Th2 cytokines.

### CD4^+^ Cell Depletion Inhibits Fibrosis and Improves Lymphatic Function

T cell inflammatory reactions have been shown to play critical roles in the regulation of fibrosis in a variety of fibroproliferative disorders both clinically and experimentally. [22] Our group has previously shown that fibrosis is a critical regulator of lymphatic function and lymphatic regeneration [14], [15], [23], [24] and fibrosis is a clinical histopathologic hallmark of lymphedema. [25] Therefore, in these experiments we sought to determine if CD4 or CD8 cell inflammation contributes to fibrosis and lymphatic dysfunction in the mouse tail model of lymphedema.

**Figure 5:**
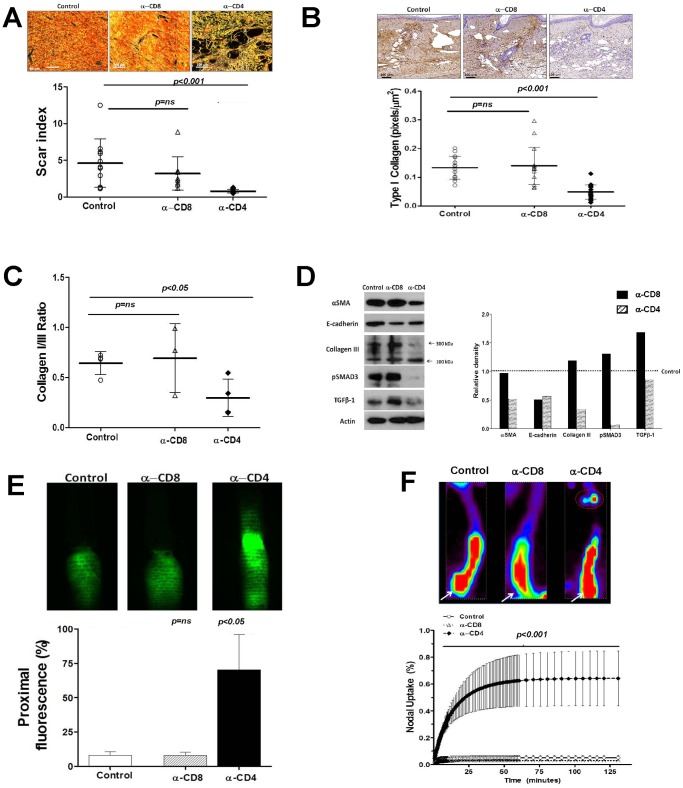
CD4+ cell depletion decreases fibrosis and improves lymphatic function. A. Scar index analysis (below) and representative photomicrographs of polarized light microscopic views (above) in control, CD8+, and CD4+ depleted animals (n = 5–7 per group) 6 weeks after surgery. **B.** Representative photomicrographs of type I collagen immunohistochemistry (above) and calculation of type I collagen staining in the dermis (positive pixels/mm^2^; below) in control, CD8+, and CD4+ depleted animals 6 weeks after surgery. **C.** Calculation of type I:type III collagen staining ratio in tail tissue sections from control, CD8+, and CD4+ depleted mice 6 weeks after surgery. **D.** Representative (of triplicate experiments) western blot analysis of a-sma, E-cadherin, type III collagen, pSMAD, and TGF-B1 in protein lysates obtained from tail tissues of control, CD8+, and CD4+ cell depleted animals 6 weeks after surgery. Quantification of band density relative to controls (arbitrarily set at 1 and represented by dotted line) is shown to the right. **E.** Representative microlymphangiography (upper) and quantification of tissue florescence proximal to the tail wound (ratio of proximal to distal florescence) in control, CD8+, and CD4+ depleted mice 6 weeks after surgery. Note crossing of the tail wound by florescent marker only in CD4+ depleted mice. **F.** Lymphoscintigraphy and sacral lymph node uptake in control, CD8+, and CD4+ depleted mice 6 weeks after surgery. Representative heat map is shown to the right (white arrow = injection site; red circle = sacral lymph nodes).

Interestingly, we found that depletion of CD4+ cells resulted in a marked decrease in dermal fibrosis as reflected by collagen deposition and organization (scar index; **Figure 5A, B**). CD4 depleted animals demonstrated 6.8 fold decrease in dermal scar index as compared with controls. In contrast, CD8 depleted animals demonstrated non-significant decreases in scar index as compared with controls. These findings were confirmed by type I collagen immunohistochemistry demonstrating a 2.8 fold decrease in type I collagen deposition in the dermis of CD4 depleted animals (**Figure 5B**). Furthermore, the ratio of type I:III collagen was significantly reduced (2.3 fold) in CD4-depleted animals, a finding consistent with decreased extracellular matrix deposition and fibrosis **(** **Figure 5C**).

Western blot analysis of whole tissue lysates from control, CD4 or CD8 depleted animals similarly revealed reductions in extracellular matrix markers consistent with decreased fibrosis (**Figure 5D**). For example, the expression of a-sma, E-cadherin, and type III collagen was decreased by 2–3 fold in CD4 depleted animals as compared with controls (**Figure 5D**). Similarly, the expression of phosphorylated SMAD-3 (14-fold) and TGF-B1 (1.4 fold) were also markedly decreased in CD4 depleted animals. This is important since we have previously shown that activation of TGF-B1signaling plays an important role in tissue fibrosis in lymphedema both clinically and in our mouse-tail model. [14], [15], [24].

To determine if decreased fibrosis in CD4 depleted animals corresponds to improved lymphatic function, we analyzed lymphatic fluid transport grossly using microlymphangiography and by analyzing radioactive tracer uptake of Tc^99^ by draining tail lymph nodes. Analysis of microlymphangiography demonstrated flow of interstitial fluid across the tail scar only in CD4 depleted animals (**Figure 5E**). In addition, quantification of florescent tracer in the proximal portion of the tail demonstrated markedly increased transport of interstitial fluid in CD4 (8.8 fold) but not CD8 depleted animals as compared with controls. These findings were confirmed with lymphoscintigraphy of the sacral lymph nodes after distal tail injection with Tc^99^ demonstrating both an increased slope (i.e. more rapid lymphatic transport) and increased decay adjusted total uptake (23-fold) in the draining lymph nodes of CD4 depleted animals (**Figure 5F**).

### CD25^+^ Cell Depletion does not Improve Lymphedema or Augment Lymphatic Function

**Figure 6:**
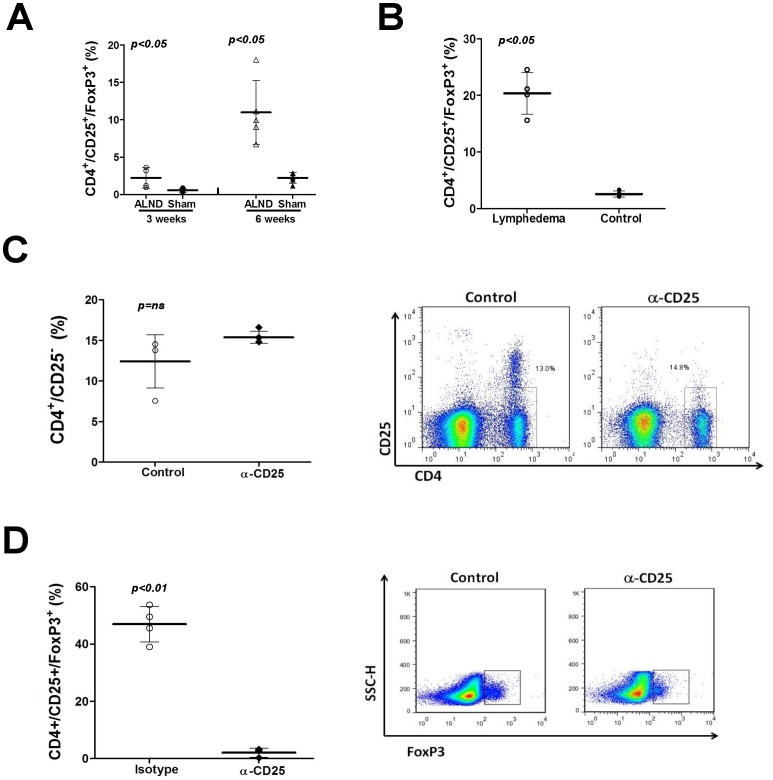
T-regulatory cell inflammation is potently increased by lymphatic fluid stasis and lymphedema. A. Flow cytometry analysis for T-regulatory (T-reg) cells in single cell suspensions prepared from upper extremity soft tissues 1.5 cm distal to the axillary wound in animals treated with axillary lymph node dissection (ALND) or axillary incision without lymphadenectomy (sham; n = 5–7/group) 3 or 6 weeks after surgery. **B.** Flow cytometry analysis for T-regulatory (T-reg) cells in single cell suspensions prepared from tail tissue 2 cm distal to the wound of lymphedema or control mice (n = 5–7/group) 6 weeks after surgery. **C.** Flow cytometry of splenic single cell suspensions for CD4+/CD25- cells after treatment with control or CD25 neutralizing antibodies. Note no significant decrease in the overall number of CD4+ cells. Representative dot plot is shown to the right. **D.** Flow cytometry of splenic single cell suspensions for CD4+/CD25+/Foxp3+ (T-regs) cells after treatment with control or CD25 neutralizing antibodies. Note no significant decrease in the overall number of CD4+ cells. Representative dot plot is shown to the right.

Because immune dysregulation and immunosuppression are a major pathological hallmark of lymphedema [6], [26] and since T regulatory (T-reg) cells are an important component of the CD4+ cell population, we next sought to determine if improvements in tail lymphedema noted in CD4 depleted animals were due to alterations in T-reg cell inflammation. In support of this hypothesis, we found that the percentage of T-reg cells (CD4+/CD25+/Foxp3+) in the upper extremity soft tissues of animals treated with axillary lymph node dissection (ALND) was significantly increased as compared with sham controls both in the early (3 week; 5.5 fold) and late (6 week; 6 fold) time points (**Figure 6A**). Similarly, we found that the percentage of T-reg cells was significantly increased (7.7 fold) in mice with tail lymphedema as compared to controls (**Figure 6B**).

In order to more specifically study the role of T-reg cells, we treated mice with CD25+ neutralizing antibodies as this treatment has been previously shown to significantly deplete T-reg cell populations and used to study T-reg responses. [27], [28] In support of this, we found that treatment with CD25 neutralizing antibodies significantly decreased the percentage of T-reg cells as compared with controls in splenic homogenates (16.8 fold; **Figure 6D**). More importantly, this treatment did not alter the percentage of CD4+/CD25- cells in these animals (**Figure 6C**).

**Figure 7:**
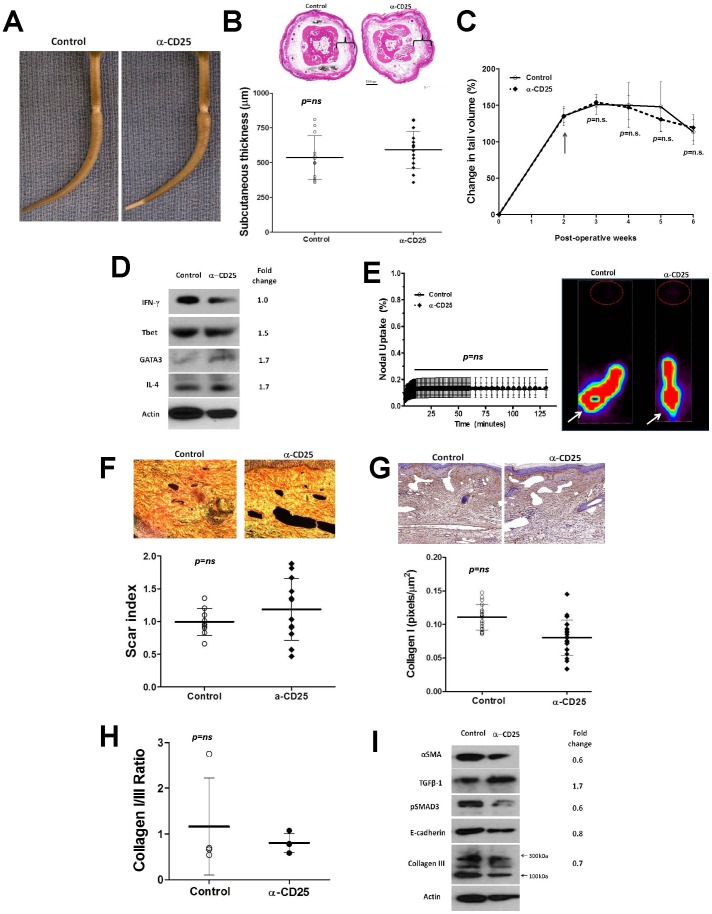
CD25^+^ cell depletion does not improve lymphedema, decrease fibrosis, or augment lymphatic function. A. Representative photograph of control or CD25+ depleted mice 6 weeks after tail superficial and deep lymphatic excision. **B.** Representative cross sectional histology and quantification of subcutaneous tissue thickness (brackets) in control and CD25+ cell depleted animals. **C.** Tail volumes in control and CD25+ cell depleted animals over the course of the experiment. CD25+ cell depletion was begun 2 weeks after surgery (arrow). **D.** Representative (of triplicate experiments) western blots from tail tissues for Th1 (IFN-y, Tbet), and Th2 (Gata-3, IL4) markers in control and CD25+ cell depleted animals 6 weeks after tail superficial and deep lymphatic excision. Quantification of band density relative to controls (fold change) is shown to the right. **E.** Lymphoscintigraphy and sacral lymph node uptake in control and CD25+ cell depleted mice 6 weeks after surgery. Representative heat map is shown to the right (white arrow = injection site; red circle = sacral lymph nodes). **F.** Scar index analysis (below) and representative photomicrographs of polarized light microscopic views (above) in control and CD25+ cell depleted animals (n = 5–7 per group) 6 weeks after surgery. **G.** Representative photomicrographs of type I collagen immunohistochemistry (above) and calculation of type I collagen staining in the dermis (positive pixels/mm^2^; below) in control and CD25+ cell depleted animals 6 weeks after surgery. **H.** Calculation of type I:type III collagen staining ratio in tail tissue sections from control and CD25+ cell depleted mice 6 weeks after surgery. **I.** Representative (of triplicate experiments) western blot analysis of a-sma, E-cadherin, type III collagen, pSMAD, and TGF-B1 in protein lysates obtained from tail tissues of control and CD25+ cell depleted animals 6 weeks after surgery. Quantification of band density relative to controls (fold change) is shown to the right.

However, despite significant T-reg cell depletion, we did not note significant differences in the gross morphology of the tail or tail volumes in animals treated with CD25 neutralizing antibodies as compared to controls **(** **Figure 7A–C** **)**. Similarly, there were no significant differences in tissue edema/subcutaneous adipose deposition, inflammatory responses, or expression of Th1/Th2 markers after CD25 depletion (**Figure 7D**). Most importantly, we found that depletion of CD25 cells did not alter fibrosis, type I/type III collagen deposition, or collagen I/III ratios (**Figure 7F–H**) and there were also no differences in the expression of fibrotic markers such as a-sma, E-cadherin, TGF-B1, p-SMAD3 (**Figure 7I**). Depletion of CD25 also failed to improve lymphatic transport across the wound or lymph node uptake of Tc^99^ (**Figure 7E**). Taken together, these findings indicate that although T-reg cell populations are increased in response to lymphatic stasis and in chronic lymphedema, the loss of these cell populations does not significantly alter the pathological manifestations (inflammation, fibrosis, lymphatic dysfunction) of this disorder.

### Loss of CD4^+^ but not CD8^+^ or CD25^+^ Cells Increases Lymphangiogenesis

**Figure 8:**
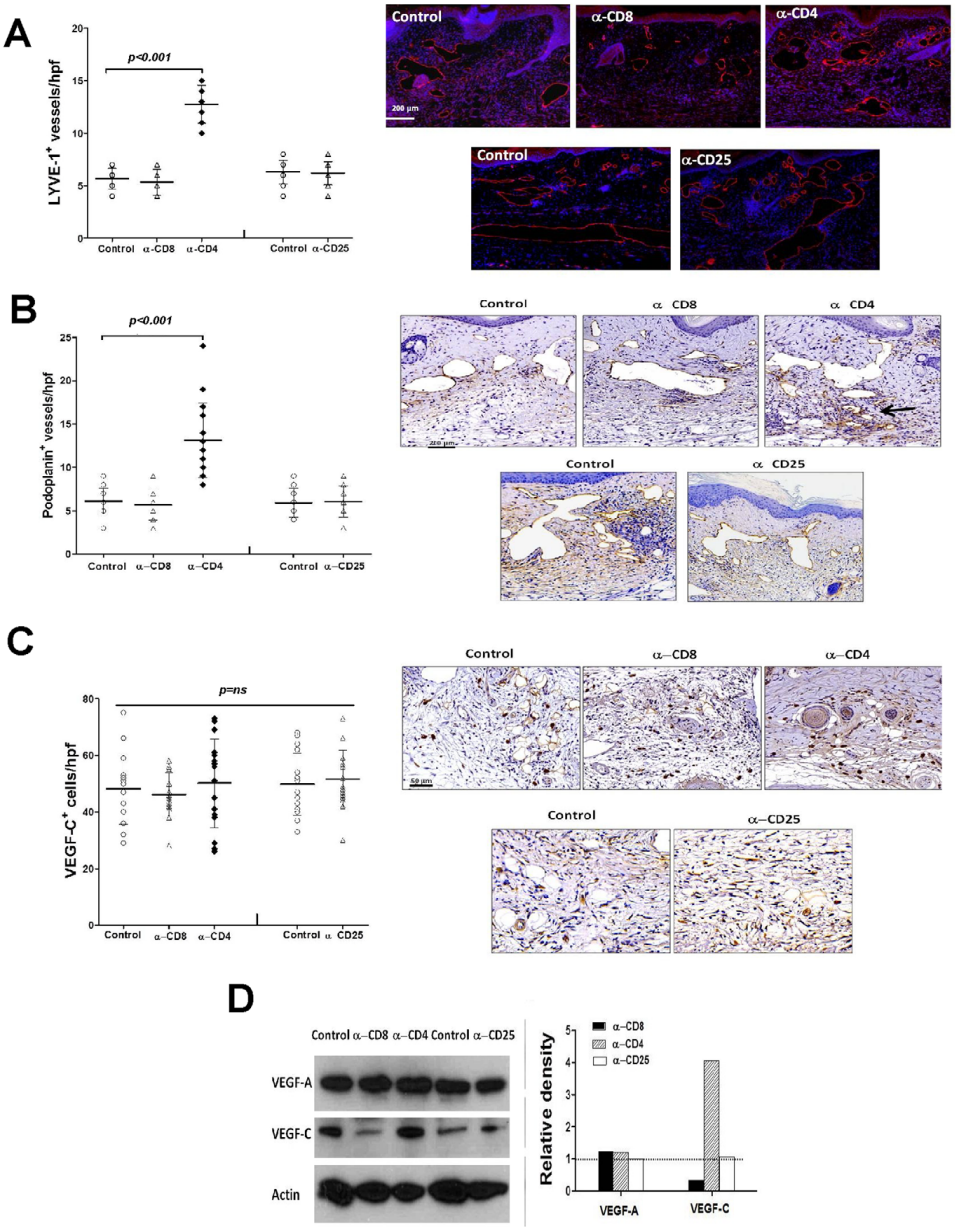
Loss of CD4^+^ but not CD8^+^ or CD25^+^ cells increases lymphangiogenesis. A. LYVE-1+ vessel counts (left) and representative figures (right) of tail sections from control, CD8+, CD4+, or CD25+ cell depleted mice 6 weeks after surgery. **B.** Podoplanin+ vessel counts (left) and representative figures (right) of tail sections from control, CD8+, CD4+, or CD25+ cell depleted mice 6 weeks after surgery. **C.** VEGF-C+ cells/high powered field (hpf) counts (left) and representative figures of VEGF-C immunohistochemistry in tail tissues from control, CD8+, CD4+, or CD25+ cell depleted animals (right) 6 weeks after surgery. **D.** Representative (of triplicate experiments) western blot analysis of VEGF-A, and VEGF-C expression in protein lysates obtained from tail tissues from control, CD8+, CD4+, or CD25+ cell depleted animals 6 weeks after surgery. Quantification of band density relative to controls (arbitrarily set at 1 and represented by dotted line) is shown to the right.

To explore the mechanisms regulating improved lymphatic function in CD4 depleted animals, we determined the effect of CD4 depletion on lymphangiogenesis and lymphangiogenic cytokine expression. When we analyzed tail tissues located distal to the zone of lymphatic disruption 6 weeks after surgery, we noted a significant increase (2.3 fold increase in LYVE-1+ vessels; 2.2 fold increase in podoplanin+ vessels) in the number lymphatic capillaries (LYVE-1+ or podoplanin+) in CD4 depleted animals as compared with controls (**Figure 8A, B**). In contrast, CD8 or CD25 depletion had little effect on this outcome. Interestingly, we did not note significant changes in the number of collecting lymphatics in any group (not shown). Western blot analysis of tail tissue lysates from these animals demonstrated little change in the expression of VEGF-A in any experimental group (**Figure 8D**). However, expression of VEGF-C was markedly increased in CD4 depleted animals (4 fold) and decreased in CD8 depleted mice (3.3 fold). Despite these changes in VEGF-A and VEGF-C protein expression in CD4 or CD8 depleted animals, we did not find significant differences in the number of VEGF-A or VEGF-C positive cells localized by immunohistochemistry suggesting that changes in total protein expression may reflect regulation of these proteins at the cellular level (**Figure 8C** **)**.

**Figure 9:**
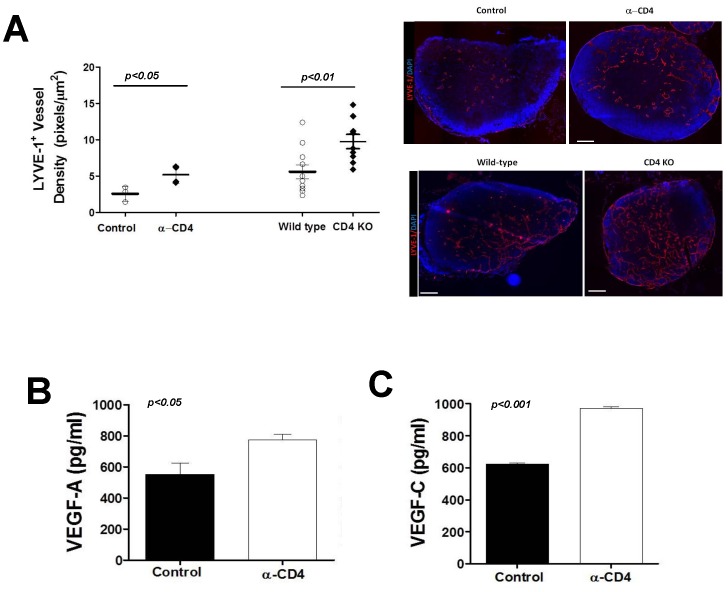
CD4+ cells regulate inflammatory lymphangiogenesis. **A.** LYVE-1+ vessel density in popliteal lymph nodes 7 days after CFA/OVA induced lymph node lymphangiogenesis in control, CD4+ cell depleted, or CD4 knockout (CD4KO) mice. Representative cross sectional histology of the lymph node (blue DAPI stain, red LYVE-1 stain) are shown to the right. **B., C.** Expression of VEGF-A (A) and VEGF-C (B) protein by ELISA in popliteal lymph nodes harvested 7 days after CFA/OVA induced lymph node lymphangiogenesis in control or CD4 depleted mice.

We used an inflammatory lymph node model in order to further explore the effects of CD4 cell depletion on acute inflammatory lymphangiogenesis since this model avoids changes that may be secondary to wound healing effects. In confirmation of our tail model observations, we found that depletion of CD4+ cells markedly increased LYVE-1+ vessel density 5–7 days after hind limb CFA/OVA injection as compared with controls (**Figure 9A** **)**. We found similar results when we repeated these experiments in transgenic mice deficient in CD4+ cells (CD4KO). In addition, we found modest though significant increases in the expression of both VEGF-A (1.3 fold) and VEGF-C (1.6 fold) protein in the lymph nodes of CD4 depleted animals as compared with controls (**Figure 9B, C**).

## Discussion

In the current study, consistent with previous reports, we found that chronic lymphedema results in tissue inflammation. Using multi-color flow cytometry to characterize the cellular populations in tissue digests, we found that lymphedema in the mouse-tail model results in significant alterations in the populations of inflammatory cells that are present. More specifically, unlike previously published studies, the use of multiple cell surface antigens enabled us to identify specific cell populations and demonstrated that the percentage of mature T-helper cells, T-regulatory cells, neutrophils, macrophages, and dendritic cells were all significantly elevated in lymphedematous tissues as compared with surgical controls. Our findings are supported by those reported by Galkowska and Olszewsk using Giemsa staining of cell smears on lymphatic fluid collected from dogs with chronic lymphedema or sham controls demonstrating significantly increased numbers of lymphocytes and veiled cells (dendritic cells) in animals with chronic lymphedema. [7] In a later study on patients with lymphedema, these same authors used histological stains and single antigen immunohistochemistry to characterize inflammatory cells populations in patients with long standing lymphedema versus normal skin and found that lymphedema patients had qualitatively more lymphocytes than controls. [5] The findings of our study are also supported by microarray studies in the mouse-tail model demonstrating acute increases in gene families regulating acute inflammation and immune responses. [16] Taken together, our findings and these previous studies support the hypothesis that lymphedema results in a mixed inflammatory response including a prominent lymphocyte component.

In order to determine how acute, low-grade lymphatic stasis resulting from lymph node resection regulates inflammatory responses, we analyzed single cell suspensions from the upper arm area of mice treated with axillary lymphadenectomy (ALND) versus axillary incision without lymph node removal 3 or 6 weeks after surgery. Interestingly, we found that ALND, similar to tail lymphedema, resulted in a persistent and significant increase in tissue inflammation even as long as 6 weeks after surgery. More importantly, we found that similar to tail lymphedema, inflammatory responses to ALND included a prominent increase in the percentage of a variety of T lymphocytes (T-helper, T-cytotoxic, Treg, NKT). However, in contrast to the inflammatory changes in the tail model, we found that ALND had only modest, and largely insignificant effects on macrophages, monocytes, and dendritic cells suggesting that T cell inflammatory responses precede and possibly contribute to the pathology of lymphedema.

We next explored the hypothesis that inflammatory responses contribute to the pathology of lymphedema and regulate, either directly or indirectly, tissue edema, adipose deposition, fibrosis, and lymphatic dysfunction. We found that depletion of CD4+ but not CD8+ or CD25+ cells resulted in marked improvements in lymphedema and decreased tail swelling, adipose tissue deposition, and fibrosis. In addition, only depletion of CD4+ cells significantly decreased tissue inflammation (CD45+ cell infiltration) and expression of Th1 and Th2 markers including IFN-y, T-Bet, and GATA-3. The hypothesis that CD4+ cell inflammation contributes to the pathology of lymphedema is supported by our previous studies demonstrating that blockade of TGF-B1 activation in the mouse tail model significantly improves lymphedema and is associated with markedly decreased tissue inflammation, CD4+ cell infiltration, and expression of Th1/Th2 markers. [14] Similarly, our hypothesis is supported by our previous studies demonstrating that mice deficient in all T cells (nude mice) have significantly decreased tail lymphedema, fibrosis, and lymphatic dysfunction as compared with wild-type littermates. [21] The hypothesis that inflammation contributes to the pathology of lymphedema is also supported by previous studies by Rockson and colleagues demonstrating that inhibition of generalized inflammatory responses using ketoprofen, a non-steroidal anti-inflammatory medication, markedly improves lymphatic function and decreases lymphedema in the mouse tail model. [29] Finally, the hypothesis that CD4+ cells either directly or indirectly contribute to the initiation and progression of lymphedema is supported by the fact that these responses have been implicated in the pathology of obesity including metabolic dysfunction and adipose deposition. [10], [30] These latter effects are important since a defining feature of end stage lymphedema is adipose deposition.

In order to determine how CD4+ cell inflammation contributes to pathology of lymphedema, we analyzed tissue fibrosis and regulators of extracellular matrix deposition. Interestingly, and consistent with our previous observations, [14] we found that depletion of CD4+ cells markedly inhibited tissue fibrosis in the regions of the tail exposed to chronic lymphatic stasis as compared with control animals or mice depleted of CD8+ or CD25+ cells. CD4+ depleted mice had significantly decreased scar index (a measure of type I collagen deposition and organization), decreased type I collagen staining by immunohistochemistry, and a decreased ratio of type I/IIII collagen deposition. These findings are important as we have previously shown that fibrosis is a significant regulator of lymphatic function. For example, we have previously shown that inhibition of radiation-induced fibrosis markedly improves lymphatic function. [24] Similarly, we have previously shown that clinical lymphedema specimens have increased expression of the profibrotic regulator TGF-B1 and that inhibition of TGF-Bsignaling either locally or systemically decreases tissue fibrosis and markedly improves lymphatic function in the mouse tail model. [14] Our current studies, also suggest that regulation of fibrosis by T cell inflammatory reactions interact with TGF-B signaling since we found that depletion of CD4+ cells markedly decreased the expression of TGF-B1 and its downstream mediator pSMAD-3.This finding is consistent with our previous study demonstrating that inhibition of TGF-B1 markedly decreases T cell inflammation and that depletion of CD3+ cells (all T cell subsets) decreased TGF-1 expression. [14] Taken together, these findings suggest that CD4+ cells regulate lymphatic function in chronic lymphedema at least in part by regulating tissue fibrosis.

Recent studies by our lab and others have also shown that T cells are independent and important regulators of lymphangiogenesis. For example, we have previously shown that nude mice (lacking all T cells) have significantly improved lymphatic function and markedly decreased expression of anti-lymphangiogenic cytokines such as TGF-B1, IFN-y, and endostatin. [21] Similarly, Koh and colleagues have shown in recent elegant studies that T cells regulate lymph node lymphangiogenesis at least in part by expressing IFN-y. [11] Finally, we have previously shown that CD4+ cell inflammation is necessary for hypoxia inducible factor-1 alpha (HIF-1a) stabilization and regulation of VEGF-A/C expression in chronic lymphedema and in inflammatory lymphangiogenesis. [19] The findings of our current study are consistent with these results as we found that depletion of CD4+ but not CD8+ or CD25+ cells markedly increased lymphangiogenesis in the lymphedematous regions of the tail. Similarly, we found that depletion of CD4+ cells or inflammation in transgenic mice lacking CD4+ cells markedly increased lymphatic vessels density in draining lymph nodes and was associated with increased expression of VEGF-A and VEGF-C. Our findings complement and add to the work of Koh and colleagues since unlike the former study we show that CD4+ cells (rather than T cells in general) are important regulators of lymphangiogenesis during wound repair and that these effects likely involve regulation of both pro- and anti-lymphangiogenic pathways. Future studies will be required to determine precisely how the balance between pro- and anti-lymphangiogenic pathways regulates lymphatic repair and regeneration during wound healing and in physiologic inflammation.

An interesting finding of our study was the massive increase in the number of T-reg cells in response to lymphatic stasis both after ALND or in response to tail lymphedema. We did not observe significant changes in pathological findings of lymphedema after depletion of CD25+ cells (i.e. fibrosis, lymphatic function, adipose tissue deposition, tail volumes) suggesting that T-regulatory cells may play additional or distinct roles in lymphatic stasis. Indeed, increased numbers of T-reg cells may simply represent a homeostatic response to the chronic inflammation induced by lymphatic stasis. This response may be responsible for immune disturbances in patients with lymphedema such as increased risk of infections (lymphangitis and cellulitis) and inability to mount immune responses or acquire immunity to vaccines when administered in the lymphedematous limb. Similarly, our findings provide a rationale for previous reports demonstrating improved allograft skin graft survival after ligation of afferent lymphatics and delayed rejection of allograft tumors in animal models. [7], [31] Additional studies are clearly required and will be performed in future reports to determine the role of T-reg cells in the pathology of lymphedema.

In conclusion, in the current study we have characterized the cellular immune response to lymphatic fluid stasis resulting from lymph node dissection and chronic lymphedema using mouse models. In addition, we have shown that depletion of CD4+ cells, but not CD8+ or CD25+ cells markedly decreases that pathological findings of lymphedema including swelling, fibrosis, adipose deposition, and lymphatic dysfunction. Although CD8+ T cells do not appear to centrally regulate the pathophysiology of lymphedema, they may play a role in other aspects of lymphatic fluid stasis and dysfunction. In addition, we have shown that CD4+ cells play an important role in the regulation of lymphangiogenesis in lymphedema and inflammatory lymphangiogenesis. Finally, we have shown that lymphatic stasis results in increased number of T-regulator cells, however, the precise role that these cells play in the regulation of the pathology of lymphedema remains unknown.
