# Correction: CD4+ Cells Regulate Fibrosis and Lymphangiogenesis in Response to Lymphatic Fluid Stasis

**DOI:** 10.1371/annotation/158f757f-7d51-40b8-a3a4-550811bf0267

**Published:** 2013-05-24

**Authors:** Jamie C. Zampell, Alan Yan, Sonia Elhadad, Tomer Avraham, Evan Weitman, Babak J. Mehrara

There was an error in the Funding statement.

The correct version of the statement is: This study was supported by a Plastic Surgery Education Foundation Research Fellowship: “Mechanisms regulating fibrosis in chronic lymphedema” (Award: $50,000; PI: Jamie Zampell; 100% Effort; Mentor: Babak J. Mehrara, M.D.). Research reported in this publication was supported by the National Heart, Lung, And Blood Institute of the National Institutes of Health under Award Number R01HL111130. The content is solely the responsibility of the authors and does not necessarily represent the official views of the National Institutes of Health. The funders had no role in study design, data collection and analysis, decision to publish, or preparation of the manuscript.

